# Antipsychotics in the Treatment of Children and Adolescents with Anorexia Nervosa: A Systematic Review

**DOI:** 10.3390/biomedicines10123167

**Published:** 2022-12-07

**Authors:** Jacopo Pruccoli, Luca Bergonzini, Angela La Tempa, Antonia Parmeggiani

**Affiliations:** 1 IRCCS Istituto Delle Scienze Neurologiche di Bologna, UOC Neuropsichiatria Dell’Età Pediatrica, Centro Regionale per i Disturbi Della Nutrizione e dell’Alimentazione in età Evolutiva, 40138 Bologna, Italy; 2 Dipartimento di Scienze Mediche e Chirurgiche (DIMEC), Università di Bologna, 40138 Bologna, Italy

**Keywords:** antipsychotics, children and adolescents, anorexia nervosa, feeding and eating disorders

## Abstract

Evidence about the use of pharmacologic agents in the treatment of Anorexia Nervosa (AN) is lacking, especially in childhood and adolescence. A systematic scoping review was conducted to outline current literature evidence about the use of antipsychotics in this population. A total of 499 studies were identified with the initial search, and 28 of these studies were selected regarding the use of olanzapine (n = 13), risperidone (n = 4), aripiprazole (n = 3), chlorpromazine (n = 3), pimozide (n = 1) clotiapine (n = 1) and multiple antipsychotics (n = 3) in these patients. Overall, major side effects were reported infrequently; improvements in psychopathology and weight measures have been suggested in the majority of the considered studies. Nonetheless, the lack of RCT or good-quality studies strongly limits the generalizability of results in clinical practice.

## 1. Introduction

Anorexia Nervosa (AN) represents a mental health condition defined by a reduced energy intake causing low body weight, fear of becoming fat or gaining weight, and a disturbance of body image [1] The Fifth edition of the Diagnostic and Statistical Manual of Mental Disorders (DSM-5) reports two possible AN subtypes: restrictive AN (ANR), in which low body weight is reached via dietary restriction and physical hyperactivity, and binge/purging AN (ANBP), characterized by recurrent episodes of purging behaviors and binge eating [1].

Epidemiological studies on AN document a recent increase in its incidence. This could be partially associated with the development of less strict diagnostic criteria, an higher public awareness of the phenomenon of eating disorders in general, and an improved ability in clinicians to provide a clear diagnosis. Incidence rates in females may reach their peaks at the age of 15 years [2]. A direct impact of body shaming and social media on the development of AN and Feeding and Eating Disorders (FED) in general has been recently documented [3,4,5].

Clinicians and researchers have assessed the potential impact of psychological, pharmacological, and nutritional interventions to treat individuals with AN. International guidelines identify psychological and behavioral interventions as the mainstay of the treatment of AN, together with nutritional assistance, preferably provided in an outpatient setting [6]. In the case of individuals with severe symptoms, day hospital or inpatient treatment settings may be considered [7].

Psychological interventions designed for the treatment of AN in adults include Cognitive Behavioral Therapy–Enhanced (CBT-E), the Maudsley Anorexia Nervosa Therapy for Adults (MANTRA), Specialist Supportive Clinical Management (SSCM), and Focal Psychodynamic Therapy (FPT). The family-based treatment (FBT) model has been specifically determined as a first-line intervention for children and adolescents with AN, despite treatment modalities adopted in adults having a role in this age group as well [7,8].

Pharmacological interventions have a limited role in the management of AN, especially in children and adolescents, mainly restricted to the management of psychiatric comorbidities. No single medication has been approved for the treatment of core symptoms of AN, and drugs should not be offered as an intervention to cure AN [7,8,9,10].

The current literature lacks studies documenting the role of psychopharmacological interventions in the management of children and adolescents with AN. Despite small to medium-sized Randomized Controlled Trials (RCT) have been published on the use of specific drugs in this population, particularly in mixed-age groups, including children and adolescents aged over 18 years, the results of these studies have not provided clear support for efficacy [8,11].

Given the complex psychopathology of some of the beliefs around body image, weight, and food, together with rigidity, obsession, and anxiety which usually characterize individuals with AN, some researchers and authors have proposed antipsychotic medications as potential therapeutic agents in this condition. Initial research on first-generation antipsychotic agents found few changes in weight or behavioral symptoms of AN. More recently, there has been increased interest in second-generation or atypical antipsychotic agents, including olanzapine [7].

Tolerability issues have hampered the use of antipsychotics in the treatment of AN and FED in general. Antipsychotics have been associated with metabolic side effects and altered eating behaviors, including increased fasting glucose and insulin levels [12], binge eating [13], and dysphagia [14].

A few researchers, finally, have reported the frequency of use of antipsychotics in individuals with AN at the developmental age, highlighting the need for clinical practice guidelines and systematic reviews in this field [15].

The present review aims to outline current literature evidence about the use of antipsychotics in AN, evaluating possible effects upon psychopathology, weight gain, and tolerability in children and adolescents.

## 2. Materials and Methods

### 2.1. Protocol for the Present Review

This systematic review was performed in November 2022 by browsing the following databases: Medline (PubMed), Cochrane Library, and Clinicaltrials.gov. The research was enhanced by searching the most relevant websites of guidelines and clearinghouses and the most important textbooks sites. The Preferred Reporting Items for Systematic Reviews and Meta-Analyses guidelines (PRISMA) flowchart was used to determine the transparent exclusion of published literature for defined reasons. Pertinent selected works were tabulated concerning study design, number, and type of participants, type of study, and main results.

Inclusion criteria:

(1) Date: published between January 1950 and November 2022.
(2) Population:
  - Children and adolescents (<18 years); if multiple age ranges are included, separated data for children and adolescents for the considered variables were required;
  - Patients with a diagnosis of AN;
  - At least one patient treated with antipsychotics, including both first- (FGA) and second-generation antipsychotics (SGA): amisulpride, aripiprazole, asenapine, blonanserin, brexpiprazole, cariprazine, iloperidone, lumateperone, lurasidone, olanzapine, paliperidone, pimavanserin, quetiapine, risperidone, ziprasidone, chlorpromazine, flupentixol, fluphenazine, haloperidol, pericyazine, perphenazine, pimozide, pipotiazine, sulpiride, trifluoperazine, zuclopenthixol.
(3) Language: English.
(4) Study Design: RCT, cohort studies, cross-sectional studies, retrospective studies, and case reports.
(5) Outcomes: reporting all these three categories (at least one element per outcome):
  - clinical use (starting dosage; maximum dosage; average dosage; duration of treatment);
  - clinical outcomes (FED-related psychopathology; weight-related variables; duration of hospitalization);
  - safety-related outcomes (Adverse Drug Reactions-ADR; subjective tolerance).

Exclusion: (1) Population: other than humans. (2) Language: other than English. (3) Study design: descriptive studies, reviews, protocols. (4) Outcomes: unreported.

Given the scoping nature of this systematic review, as well as the scarce evidence existing in the field, both controlled (included patients not treated with antipsychotics) and uncontrolled studies were included.

Search string: (((Child* OR Adolescent* OR Teen* OR Young* OR Youth* OR Pediatr* OR infant) AND (Anorexia Nervosa OR Eating and Feeding Disorder* OR Feeding and Eating Disorder* OR Eating Disorder*) AND (Antipsychotic* OR dopamine antagonist* OR neuroleptic* OR Amisulpride OR Aripiprazole OR Asenapine OR Blonanserin OR Brexpiprazole OR Cariprazine OR Iloperidone OR Lumateperone OR Lurasidone OR Olanzapine OR Paliperidone OR Pimavanserin OR Quetiapine OR Risperidone OR Ziprasidone OR Chlorpromazine OR Flupentixol OR Fluphenazine OR Haloperidol OR Pericyazine OR Perphenazine OR Pimozide OR Pipotiazine OR Sulpiride OR Trifluoperazine OR Zuclopenthixol)) AND (english [Language])) AND (“1950” [Date-Publication]: “2022” [Date-Publication]).

Given the scarcity of randomized studies in this field (Couturier et al., 2020), the Newcastle-Ottawa Scale (NOS) quality assessment [16], a quality score for non-randomized studies, was adopted.

This quality score includes two different scoring subsystems:

− A system for case-control studies, scoring the three domains of “selection” (adequateness of the case definition, representativeness of the cases, selection of controls, definition of controls), “comparability” (comparability of cases and controls based on the design or analysis), and “exposure” (ascertainment of exposure, same method of ascertainment for cases and controls, Non-Response rate);
− A system for cohort studies, scoring the three domains of “selection” (representativeness of the exposed cohort, selection of the non-exposed cohort, ascertainment of exposure, demonstration that outcome of interest was not present at the start of the study), “comparability” (comparability of cohorts based on the design or analysis), and “outcome” (assessment of outcome, follow-up long enough for outcomes to occur, adequacy of follow up of cohorts) [16].

The obtained results were converted into a quality scale (good/fair/poor) according to the dedicated converting system [17]. Case reports received no quality assessment.

A total of 499 original articles were identified with the initial search and checked by two independent investigators, and disagreements among reviewers were resolved through a mediator.

The identified records were screened for appropriateness by two independent reviewers. At this phase, records were excluded:

− If published in a language different from English;
− If the nature of the study was clearly reported as a narrative review, a systematic review, a study protocol, or any other study type other than those admitted (RCT, cohort studies, cross-sectional studies, retrospective studies, and case reports);
− If the included population was clearly reported as composed only of animals other than humans.

The full texts of the records which were retained after the initial screening were sought for retrieval. Then, the obtained reports were assessed for eligibility by two independent reviewers. At this phase, records were excluded:

− If one of the exclusion criteria was still met after the appropriateness screening (population: other than humans; language: other than English; study design: descriptive studies, reviews, protocols);
− If children and adolescents (aged < 18 years) were not included in the study, or if a mixed group of children/adolescents and adults was included, but separated results for the two age groups were not provided;
− If no individual was assigned a diagnosis of AN;
− If no individual was treated with an antipsychotic;
− If the study did not report in any way the required clinical outcomes. No report of ADR in the presence of a described clinical follow-up was accepted; in this case, studies were assigned a “no ADR reported” outcome.

The eligible studies were included in the systematic review and categorized on an informatic spreadsheet. Then, a risk-of-bias and quality assessment was performed according to the described procedures by two independent reviewers. Given the scarce nature of the existing evidence, mainly including case reports and small observational studies, no study was excluded based on the received risk-of-bias score.

**Figure 1:**
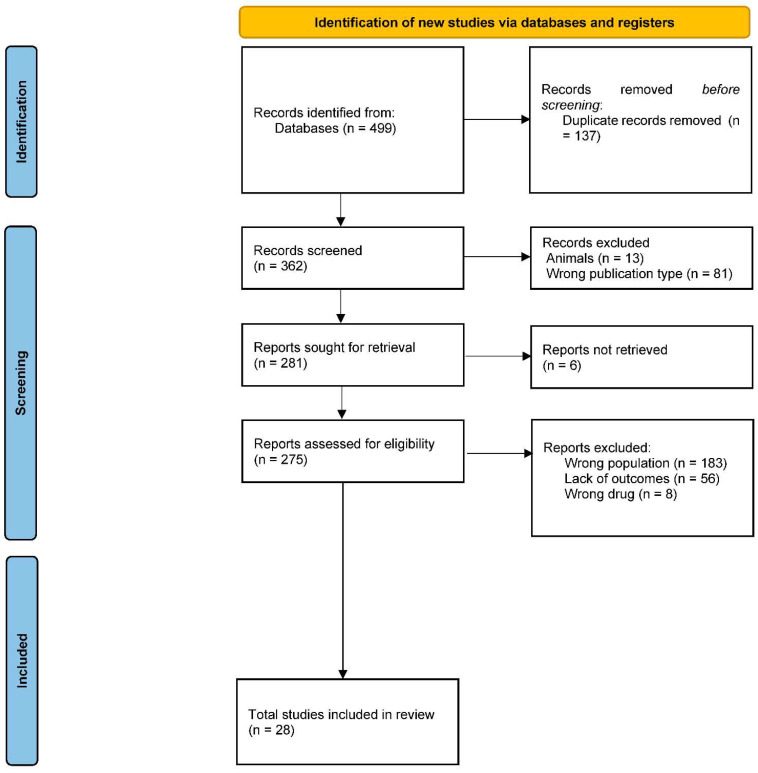
PRISMA diagram.

Twenty-eight studies were included in the final analysis. The flow chart of the study is reported in Figure 1.

### 2.2. Statistical Analysis

Given the nature of the included studies, mainly case reports or observational studies reporting the use of one or more concurrent drugs on a small number of patients, no meta-analysis was performed. All the data were presented on a descriptive basis, and results were provided for the overall group of research. Then, data were divided into gender (males and females) and age (children and adolescents) groups. Age groups were classified according to the system consistently adopted in the international literature of AN in the developmental age (children: age < 14 years; adolescents: ≥14 years) [18,19]. In these tables, studies with mixed gender or aged groups were included only if distinct results for the different groups (or patients belonging to different groups) were reported.

## 3. Results

Twenty-eight studies were included in the systematic review. The summary of the results is reported in Table 1. The quality assessment of the included study is reported in Table 2. The available evidence for patients of different gender or age groups is reported in Table 3 and Table 4.

### 3.1. Olanzapine

The literature research highlighted 13 studies regarding the use of olanzapine in the treatment of children and adolescents with AN (five observational studies and eight case reports).

Spettigue and colleagues [20] describe a non-randomized open-label trial comparing twenty-two participants who took olanzapine (medication group) and ten participants who did not (comparison group). Participants in the medication group demonstrated a higher rate of weight gain compared to those who did not receive olanzapine (*p* = 0.012).

Mild side effects, such as headache, constipation, muscle dizziness, stiffness, somnolence, and dry mouth were reported by people treated with olanzapine; overall, seven participants (31.8%) discontinued the drug due to side effects [20].

In a prospective study, Leggero and colleagues evaluated the effects of a low-dose olanzapine monotherapy (mean dose 4.13 mg/day) in thirteen female patients relying upon standardized measures at baseline and after 1 and 6 months of therapy. Patients showed a significant improvement in weight, global functioning, eating attitudes, anxious-depressive symptoms, and hyperactivity. At the end of the 6-month follow-up, 7 patients were classified as responders since they showed an improvement of at least 50% in the Eating Attitude Test-26 (EAT-26) results. Hyperactivity was the only measure that improved significantly in responders, but not in non-responses, according to the Structured Inventory for Anorexic and Bulimic Eating Disorders (SIAB-EX) [22].

Pruccoli and collaborators carried out an observational naturalistic case-control study about the use and tolerability of low-dose olanzapine in a multidisciplinary hospital intervention for adolescents with AN. Three groups with AN were treated with low-dose, high-dose, or no olanzapine and were compared. Psychopathology was assessed through the administration of questionnaires at hospital admission and discharge. Individuals treated with low-dose olanzapine and those treated with no SGA reported better outcomes on depressive symptoms than those treated with full-dose olanzapine. The treatment with olanzapine was well tolerated by 86.4% of the patients on low or full dose; mild elevation of total cholesterol levels or transaminases, somnolence, reduction of blood pressure, and elongation of the PR interval were among the side effects reported by the remaining population [24].

Norris and colleagues conducted a retrospective study and compared medical measures and treatment courses in a sample of adolescent women with AN treated with olanzapine with a matched cohort that did not receive olanzapine or any other SGA. Patients treated with olanzapine displayed greater evidence of psychopathology and medical compromise at the time of the first assessment. The rate of weight gain was not statistically different between groups when olanzapine was started during inpatient admissions. Medication effects on eating disorder cognitions could not be assessed, given the presence of multiple confounders. Side effects included sedation and dyslipidemia in 56% of patients [21].

In a retrospective study, Rossi and collaborators evaluated the efficacy and safety of pharmacotherapy in 19 anorexic preadolescents and adolescents who were referred to a specialist psychiatry unit for the first time. Antidepressants accounted for 75.7% of the prescriptions, followed by antipsychotic medications (21.6%), of whom 4 patients have been treated with haloperidol, 3 patients with olanzapine, and 1 with risperidone. Based on CGI assessment, 75% of patients treated with antipsychotics showed an improvement in their eating behavior and psychological status, with BMI increasing in all patients but one; drowsiness has been the most frequent side effect reported [45].

Dennis and colleagues described side effects and psychopathological changes in five adolescent females with AN treated with olanzapine. Four patients reported a decrease in anxious feelings, two patients reported incremented sedation. Weight increase was documented only in two out of five patients [23].

Boachie and colleagues reported anxiety reduction, sleep improvement, and weight gain with no side effects in a case series exploring the use of olanzapine as an adjunctive treatment in four females with AN in a pediatric inpatient setting [25].

Mehler and collaborators found a reduction of delusional thoughts and weight concerns in five female adolescents with chronic AN who received olanzapine. No significant side effects were reported; one patient experienced an episode of binge eating due to increased appetite. Eventually, all patients experienced weight gain [26].

Ercan and colleagues reported the case of a 15-year-old teenager with AN who required admission to the Intensive Care Unit due to extreme weight loss and agitation. Olanzapine was started in the ICU; during the 16 weeks of stay, remission of psychiatric symptoms (such as irritability, compulsive thoughts about body weight, food, and physical activity) occurred. No side effects were mentioned [27].

La Via et al. described the case of a 15-year-old female teenager with a 5-year history of AN, purging type, who started a pharmacological trial with olanzapine. During her hospitalization, a reduction of anxiety and agitation and a significant gain in weight was reported. Olanzapine was tolerated, and only mild sedation was documented [29].

Duvvuri and collaborators analyzed the differential weight restoration in identical twins with AN, treated with olanzapine (twin B) or fluoxetine (twin A). Both twins attended the same family therapy sessions. In 9 months, eating disorder preoccupations or rituals reduced. Some differences were noted: the twin treated with olanzapine started expressing hunger at mealtimes, and she also regained her normal eating pattern; in the end, twin B was remarkably weight restored at 99.9% IBW, while twin A had yet to weight restore at 84.4% IBW [30].

Ayyildiz and colleagues reported the case of a 17-year-old male with AN who experienced an NMS after two days of treatment with a low dose of olanzapine. The symptoms consisted of high fever (max 40 °C), leukocytosis, elevated levels of creatine kinase (CPK), and fluctuation in his mental state. Although the drug was discontinued, NMS developed again after 30 days [31].

Tateno and Collaborators described the case of a 17-year-old girl with a 1-year history of AN. After an initial refusal, she agreed to start low-dose olanzapine. Approximately 6 weeks later, weight gain without adverse effects began, and 5 months later, her weight reached the standard. Indeed, also her rigidity and tendency to impose routines improved [32].

### 3.2. Risperidone

Four case reports analyzed the role of risperidone in the treatment of AN in childhood and adolescence.

Kracke and Collaborators described four years follow-up of an adolescent female with AN, restrictive subtype. After three months of residential treatment and two years of pharmacological treatment with SSRI, she got no significant results. Then, she agreed to begin a low dose of risperidone. During the treatment with low-dose risperidone, she benefited from decreased rigid thinking, weight gain, and resolution of secondary amenorrhea without medication side effects [33].

Umehara and Colleagues presented the case of a 10-year-old boy with a 1-year history of a restrictive eating disorder with agitation during meals. He started oral risperidone, with remarkable improvement in his eating behavior and weight. The drug was discontinued in two months due to the patient’s belief that risperidone was making him fat. A second successful attempt was done with a risperidone long-acting injection (RLAI) [34].

J-Newman-Toker reported the case of a 12-year-old girl with a 2-year history of a restrictive eating disorder. With the assumption of risperidone, she reported feeling more cheerful and energetic, and she expressed more insight about anorexia and lowered anxiety. Furthermore, she gradually gained weight, and after 9 months, her periods came [35].

Fisman and Collaborators described the hospital treatment of a 13-year-old girl with AN and Autism who has experienced a change in eating habits for a year. During low hospitalization dose of risperidone was started, and both parents and staff observed a psychopathology improvement, more compliant behavior, and a significant weight gain. At 12 months post-discharge, her condition was stable [36].

### 3.3. Aripiprazole

Three studies (a retrospective study and two case series) analyzed the role of aripiprazole in the treatment of AN in children and adolescents.

Tahilioglu et al. reported a case series of eleven females with AN treated with aripiprazole. Improvements in obsessive eating attitudes and behaviors were found in all patients: the change in CGIS scores and BMI were statistically significant (*p* < 0.001) [40].

Frank and collaborators reported a case series of four females with severe and recurrent AN treated with aripiprazole. The patients experienced improvement in body image distortion and food avoidance behaviors, with overall global improvement in the ideal body weight. One patient experienced a drop in the white cell neutrophil count; aripiprazole was discontinued, with count normalization. After a cautious rechallenge, the neutrophil count slightly dropped and stabilized, with the patient reporting subjective improvement in the eating disorder psychopathology [38].

Recently, the same authors presented a retrospective chart review of 106 adolescents with AN. Twenty-two patients were treated with aripiprazole in a specialized eating disorder program and were compared to matched controls. The effects from the prescription were by absolute BMI values modest (6% higher in the aripiprazole group), but the BMI percentile was about 20% higher at discharge compared to the no-aripiprazole group [39].

### 3.4. Clotiapine

A retrospective chart review analyzed the role of clotiapine in the treatment of AN in childhood and adolescence. From the selection sample, only two females were treated with clotiapine. In the first patient, the treatment was withdrawn due to discomfort and fatigue within 3 days; risperidone was prescribed with a resolution of asthenia. The second case tolerated the pharmacological treatment and experienced improvement in delusional symptoms [37].

### 3.5. Pimozide

A single case report described the administration of pimozide in a 17-year-old male with AN. The author reported significant improvement within three weeks of treatment: an important weight gain and reduction in obsessive thoughts about his weight [41].

### 3.6. Chlorpromazine 

Three case reports were found about the role of chlorpromazine in children and adolescents with AN.

Foster and Kupfer analyzed the case of a 17-year-old female who was hospitalized after six months of restrictive attitude and a significant loss of weight. During her recovery, she started chlorpromazine which helped her with regaining her weight and diminishing obsessional food handling rituals [42].

Roberts and Collaborators reported the case of a 16-year-old female who started pharmacological treatment with low-dose chlorpromazine. After six weeks, her weight improved, but she had a status epilepticus due to a low level of serum sodium. Hyponatremia in this patient was most likely caused by water excess. Syndrome of inappropriate antidiuretic hormone release (SIADH) was excluded [43].

Roussonis described an 11-year-old boy with AN who gradually increased his caloric intake until he regained his premorbid weight in 90 days [44].

### 3.7. Studies including Multiple Drugs

Three studies were found about the use of multiple antipsychotic drugs for the treatment of children and adolescents with AN, a retrospective study, and two case reports.

Rossi and collaborators [45] analyzed retrospectively 19 patients with AN who received pharmacological treatment. The 21.6% were treated with an antipsychotic drug (haloperidol, olanzapine, and risperidone). Almost all the patients showed an improvement both in their eating behavior and in BMI (except one treated with haloperidol due to exacerbation of restrictive eating behavior). Furthermore, no significant side effects were reported (only 12.5% reported severe drowsiness).

Ritchie and collaborators described the case of a 15 years old female with AN who experienced a corrected QT interval (QTc) prolongation at EKG after the introduction of olanzapine. The drug was held, and she was administered risperidone after 30 days, which was promptly discontinued due to QTc prolongation. The QTc normalized within a week, but her anxiety and eating disorder preoccupation intensified, resulting in a further trial of low-dose quetiapine. The patient tolerated quetiapine with no QTc changes. Anxiety improved, and weight restoration occurred [46].

Dodig-Ćurković and colleagues reported the case of multiple pharmacological treatments in a 15-year-old girl with AN. After two attempts with SSRIs that both determined increased liver transaminase, she started sulpiride with good tolerance. During the examination, it was observed that the patient was physically and mentally exhausted, so low-dose olanzapine was added to sulpiride and gradually increased. This pharmacological combination determined a significant weight gain and improvement in psychopathology [47].

### 3.8. Other Drugs

No evidence was found for amisulpride, asenapine, blonanserin, brexpiprazole, cariprazine, iloperidone, lumateperone, lurasidone, paliperidone, pimavanserin, ziprasidone, chlorpromazine, flupentixol, fluphenazine, periciazine, perphenazine, pimozide, pipotiazine, sulpiride, tri-fluoperazine, zuclopenthixol.

## 4. Discussion

The present systematic review collects the existing data concerning the use and tolerability of antipsychotics in the treatment of children and adolescents with AN. Overall, 28 studies were included. The quality of observational studies ranged from good (1 study), fair (1 study), and poor (5 studies). Thirteen studies were case reports or case series adopting a strict case-report structure.

Thirteen papers about the use of olanzapine in the treatment of children and adolescents with AN were found in the literature: one good-quality retrospective observational cohort study [21], one fair-quality retrospective observational case-control study [24], one poor-quality non-randomized open-label trial studies [20] and one poor-quality prospective study [22]; the remaining eight papers include case series or case reports.

Only Norris and colleagues describe an absence of improvement in weight measures and psychopathology with olanzapine administration [21]. Despite this being the only included study reporting such negative evidence, the readers should consider that this also represented the only “good-quality” included study according to the above-mentioned score for non-randomized studies. Thus, the existing evidence appears not univocal, and researchers and clinicians should carefully weigh the available data before prescribing olanzapine to young subjects with AN.

In the other papers, anecdotal weight gain and psychopathology improvements were reported, especially in anxiety [20,23,24,25], depression [20,24], obsessions and compulsions [23,25,27,30].

No clear-cut, olanzapine-related adverse effects were reported by five papers [25,26,27,28,30]. A severe side effect was reported in a sole case report where the onset of Neuroleptic neuroleptic malignant syndrome after two days of treatment is described [31]. Liver enzymes [21,22] and lipid profile/cholesterol elevation [21,24], itching [23], headache [20] and sedation/somnolence [20,21,23,24,29] were reported, with only one case of QTc elongation [46]. Drug withdrawal due to side effects was rarely described [23]. No speculation about possible predictive factors of drug-related adverse effects is possible from the gathered data.

Outcome discrepancies between good and poor-quality studies about the efficacy of olanzapine in these patients hamper its role in the treatment of the disease, despite an apparent good tolerability profile; further evidence is needed in this field.

Only five case reports describe the use of risperidone in young people with AN [33,34,35,36,46]. These papers describe an overall improvement in body-weight measures and psychopathology, with good tolerability and no side effects, except for one case in which the drug was discontinued due to QTc elongation; one case of discontinued assumption due to the belief of becoming fat is described, however, the use of RLAI was unharmful [34]. Given the lack of higher-quality evidence in this field, the efficacy and tolerability of risperidone in the treatment of young patients with AN are only anecdotal, and further studies are needed to support its use in clinical practice.

The current review identified scant evidence for the use of aripiprazole in children and adolescents with AN. Two poor-quality studies [39,40] and one small case series with 4 patients [38] were included. Concerning the considered outcomes, all the studies reported an improvement in body-weight measures, while two studies showed improved psychopathology [38,39]. A good tolerability was described, with only one case of neutropenia [38]. Despite this evidence, the included studies suffered from a lack of a comparison group [40] or poor reporting of outcomes [39], or had a clear case-report nature [38]. These data suggest a potential role of aripiprazole in improving weight gain in children and adolescents with AN, with an apparent good tolerability profile. Nonetheless, the lack of RCT or good-quality studies strongly limits the generalizability of these results in clinical practice.

Currently, the included literature lacks any evidence concerning the use of clotiapine in the management of AN. Indeed, the present review included a single retrospective chart review [37]. Regarding the considered outcomes, the paper describes a scarce tolerance to pharmacological therapy in one out of two cases and an improvement of psychopathology in the patient who continues the assumption. These data do not allow us to draw reliable conclusions; further validation of this postulate is needed.

The current review identified scant evidence for the use of chlorpromazine in children and adolescents with AN. Three case reports [42,43,44] have been included where a role in regaining the weight is suggested. The scarcity of data collected, partly due to the current increased use of SGA in clinical practice, strongly limits us from accounting for chlorpromazine as a therapeutic option.

In a study reporting on the use of different antipsychotics in young patients with AN, Rossi and colleagues included 4 patients treated with haloperidol, together with 3 patients receiving olanzapine and 1 risperidone [45]. Beyond one patient reporting severe drowsiness, a worsening of the eating disorder symptoms was reported in one case treated with haloperidol. Globally, an improvement in psychopathology and weight measures was reported in most of the other cases [45]. Nonetheless, given the specific nature of the study (different antipsychotics administered to the treatment group), issues concerning the control group (treated with SSRI), and the considerably small sample, a poor quality evaluation was provided in this review to the described study, and insufficient evidence exists for the use of haloperidol in children and adolescents with AN.

## 5. Limitations and Strengths

This study has some limitations. The relatively strict inclusion criteria allowed us to retain for the final analysis only a small number of studies, excluding mixed populations of adults and adolescents without specific data on the two groups. Due to the scarce existing evidence in this field, no meta-analysis was conducted. Nonetheless, this study shows some strengths. It represents the first research to collect the existing data for all antipsychotic types, including both first- and second-generation antipsychotics. The strict inclusion criteria adopted permitted the inclusion of a relatively homogeneous group of studies.

## 6. Conclusions

In conclusion, this review systematically assesses the use and tolerability of antipsychotics for the treatment of AN in children and adolescents. One good-quality and one fair-quality study on the use of olanzapine was included and described. Poor quality evidence and case reports were obtained for the use of risperidone, aripiprazole, clotiapine, quetiapine, and haloperidol. Olanzapine presented a better quality of data when compared to the other included drugs, with evidence of improvements in psychopathology and weight measures in most of the included studies, with infrequently reported side effects. Nonetheless, given the lack of strong quality evidence, the use in children and adolescents with AN of this drug, and antipsychotics in general, remains limited, and psychological and nutritional interventions persist as the mainstay for the treatment of this condition.

## Figures and Tables

**Table 1:** Summary of the results of the included studies.

Study	Population	Intervention	Comparison	Outcome
Olanzapine				
Spettigue et al., 2018 [20]	N: 32; sex: 29 females, 3 males; mean age: 15.48; age range: 11–17.	Olanzapine Min. dose: 1.25 mg/day (range: 1.25–5 mg/day); Max dose: mean 5.28 mg/day (range: 2.5–15 mg/day); Duration: mean 55.18 days; range 16–86 days.	Eligible to switch into the medication group up until the end of week 8 in the study	Side effects/tolerability: patients tended to experience headaches and constipation more than the comparison group. They reported more muscle dizziness, stiffness, somnolence, and dry mouth; Psychopathology: decrease in depression and anxiety; decrease in self-reported ED behavior; Weight: more weight per week than those in the comparison group; Duration of hospitalization: na
Norris et al., 2011 [21]	N: 43; sex: females; mean age: 14.4; age range: 10–17.	Olanzapine Min. dose: 3.5 mg/day; Max. dose: 7.5 mg/day; Mean dose: 5.0 mg/day; Duration: 252 days;	Patients chosen by age and diagnosis at assessment, and then treatment intensity level was undertaken after assessment (i.e., outpatient care, entry into our day program, or admission to the inpatient unit).	Side effects/tolerability: sedation (more common); abnormalities in blood work (elevations in the fasting lipid profile; one had elevated liver function test); Psychopathology: no significative differences were found; Weight: no significant gain; Duration of hospitalization: na
Leggero et al., 2010 [22]	N: 13; sex: females; mean age: 13.7; age range: 9.6–16.3.	Olanzapine Min. dose: 1.25 mg/day (range: 1.25–2.5 mg/day); Max dose: mean 4 ± 3.40 mg/day (range: 3.75–12.5 mg/day); Mean dose: 4.1 ± 2.9 mg/day Duration: n.a.	/	Side effects/tolerability: An increase in liver enzymes occurred in two patients; Psychopathology: CGAS and CGI-S improved significantly. Both the CBCL Total Score and Internalizing Scale improved significantly. The EAT-26 total scores markedly improved, including Dieting, Bulimic, and Oral Control. The EDI-2 showed significant improvement only in two areas, Interoceptive Awareness, and Impulsivity; hyperactivity (according to SIAB-EX) resulted in significant improvement; Weight: BMI score improved significantly; Duration of hospitalization: na
Dennis et al., 2006 [23]	N: 5; sex: females; mean age: 15.6; age range: 12–18.	Olanzapine Min. dose: 1.25 mg/day; Max dose: 7.5 mg/day; Duration: n.a.	/	Side effects/tolerability: pt 1: mild sedation; pt 4: excessive sedation; patient 5: itching (stop assumption after 2 months); Psychopathology: pt 1: anxiety around mealtimes subsided and agitation with family decreased markedly; pt 2: improved sleep and decreased anxiety; decreased irritability and combativeness at mealtimes; pt 3: decreased worry about eating and decreased body image concern. She reported less rumination about food, fewer depressive symptoms, and decreased anxiety; pt 4: decreased anxiety. She felt calmer before meals; pt 5: no improvement was recorded; Weight: improved markedly only for two patients; Duration of hospitalization: n.a.
Pruccoli et al., 2022 [24]	N: 66; sex: 63 females, 3 males; mean age: 15.4; age range: 13–18.	Olanzapine Group 1, (low-dose) N: 37 Min. dose: mean 3.4 mg/day; Max dose: mean 4.4 mg/day; Duration: mean 132 days; Group 2 (full-dose) N: 29 Min. dose: mean 6 mg/day; Max. dose: mean 9 mg/day; Duration: mean 132 days;	Same characteristics of the cases, but not treated with antipsychotic	Side effects/tolerability: Group 1: two patients (5.4%) developed a mild elevation of total cholesterol levels; one patient (2.7%) showed elevated transaminase. Group 2: 3 patients (10.5%) presented a mild elevation of total cholesterol levels, 1 patient (3.5%) developed somnolence, 1 patient (3.5%) showed a reduction of blood pressure; Psychopathology: improvement of BUT-GSI (*p* < 0.001), BDI-II (*p* < 0.001), and SAFA-D (*p* < 0.001) for the entire sample, but group 2 experienced a significantly lower improvement in depressive measures than the other groups; Weight: significant gain. Duration of hospitalization: group 1: mean 121 days; group 2: mean 116 days.
Boachie et al., 2002 [25]	N: 4; sex: females; mean age: 11; age range: 10–12.	Olanzapine Min dose: 2.5 mg/day; Max dose: 2.5 mg/day; Duration: pt 1: n.a.; pt 2: 155 days; pt 3: 84 days; pt 4: n.a.	/	Side effects/tolerability: no ADR reported; Psychopathology: pt 1: better in sleeping, less anxious, fewer compulsive rituals, and fewer food preoccupations; pt 2: at discharge, she was not agitated, was sleeping well, was an improved relationship with others; pt 3: clinical improvement in her general and premeal anxiety levels, as well as a reduction in agitation; pt 4: improvement in her general anxiety level and agitation’s reduction; Weight: significant gain; Duration of hospitalization: pt 1, 49 days; pt 2, 70 days; pt 3, 77 days, pt 4, 120 days.
Mehler et al., 2001 [26]	N: 5; sex: females; mean age: 15.4; age range: 12–17.	Olanzapine Min. dose: 2.5 mg/day (range 2.5–10 mg/day); Max dose: 12.5 mg/day (range 5–12.5 mg/day); Duration: pt 1, 42 days; pt 2, 112 days; pt 3, 56 days; pt 4, 48 days; pt 5: na	/	Side effects/tolerability: no ADR reported. Pt 3 increased appetite with the first episode of binge eating; Psychopathology: improvement of the delusional quality of thinking, and fears about weight; Weight: significant gain; Duration of hospitalization: pt 1: 77 days, pt 2: 56 days; pt 3: 70 days; pt 4 n.a.; pt 5 n.a.
Ercan et al., 2003 [27]	N: 1; sex: female; age: 15.	Olanzapine: Min. dose: 2.5 mg/day; Max dose: 10 mg/day; Duration: 196 days;	/	Side effects/tolerability: no ADR reported; Psychopathology: remission of psychiatric symptoms (irritability, compulsive thoughts about body weight, food, and physical activity) Weight: significant gain; Duration of hospitalization: 133 days.
Dadic-Hero et al., 2009 [28]	N: 1; sex: female; age: 15.	Olanzapine Min. dose: 2.5 mg/day; Max dose: 5 mg/day; Duration: 720 days;	/	Side effects/tolerability: no ADR reported Psychopathology: na Weight: significant gain; Duration of hospitalization: 112 days.
La Via et al., 2000 [29]	N: 1; sex: female; age: 15.	Olanzapine Min. dose: 2.5 mg/day; Max dose: 15 mg/day; Duration: 180 days;	/	Side effects/tolerability: mild sedation; Psychopathology: reduction in agitation and anxiety; Weight: significant gain; Duration of hospitalization: 98 days
Duvvuri et al., 2012 [30]	N: 1; sex: female; age: 12.	Olanzapine Min. dose: 2.5 mg/day; Max dose: 3.75 mg/day; Duration: 270 days;	Identical twin, with the same diagnosis, treated with fluoxetine	Side effects/tolerability: no ADR reported Psychopathology: reduced ED preoccupations or rituals, obsessions, and compulsions; Weight: IBW restored; Duration of hospitalization: outpatient treatment.
Ayyildiz et al., 2015 [31]	N: 1; sex: male; age: 17.	Olanzapine Min dose: 5 mg/day; Duration: 2 days	/	Side effects/tolerability: NMS occurred 48 h after the first administration; Psychopathology: no improvement; Weight: no improvement; Duration of hospitalization: na.
Tateno et al., 2008 [32]	N: 1; sex: female; age: 17	Olanzapine Min. dose: 2.5 mg/day; Max. dose: 5 mg/day; Duration: na	/	Side effects/tolerability: no ADR reported; Psychopathology: abnormal eating habits gradually remitted; Weight: reached the standard; Duration of hospitalization: na.
Risperidone				
Kracke et al., 2014 [33]	N: 1; sex: female; age: 17.	Risperidone Min. dose: 0.25 mg/day; Max: dose: 0.5 mg/day; Duration: 670 days;	/	Side effects/tolerability: no ADR reported Psychopathology: improvement in rigidity during meal timing; Weight: significant gain; Duration of hospitalization: no hospitalization.
Umehara et al., 2014 [34]	N: 1; sex: male; age: 10	Risperidone Min. dose: 1 mg/day; Duration: 84 days; Risperidone long-acting injection (RLAI) Dose: 12.5 mg/2 weeks; Duration: 365 days.	/	Side effects/tolerability: he refused oral risperidone because he believed it was making him fat Psychopathology: reduction of agitation during enteral feeding; reduction of his complaints based on body distortion Weight: significant gain; Duration of hospitalization: 448 days.
Newman-Toker, 2000 [35]	N: 1; sex: female; age: 12.	Risperidone: Min. dose: 0.5 mg/day; Max dose: 1.5 mg/day; Duration: n.a.	/	Side effects/tolerability: no ADR reported; Psychopathology: cheerful, energetic, insight about anorexia, lowered anxiety Weight: BMI improvement; Duration of hospitalization: na
Fisman et al., 1996 [36]	N: 1; sex: female; age: 13.	Risperidone Min. dose: 0.5 mg/day; Duration: na	/	Side effects/tolerability: no ADR reported; Psychopathology: striking decrease in agitation and aggression, loss of paranoid ideation, more focused attention, and generally more settled and malleable behavior. Weight: significant gain; Duration of hospitalization: na.
Clotiapine				
Pruccoli et al., 2021 [37]	N: 2; sex: females; mean age: 17.5; age range: 17–18.	Clotiapine Min. dose: mean 15 mg/day (range 10–20); Max dose: 70 mg/day; Duration: 270 days;	/	Side effects/tolerability: fatigue (pt 1) which caused withdrawal in 3 days; Psychopathology: decrease of delusional symptoms, restored proper hygiene habits; Weight: n.a. Duration of hospitalization: 330 days.
Aripiprazole				
Frank et al., 2016 [38]	N: 4; sex: females; mean age: n.a.; age range: 12–17	Aripiprazole Min. dose: mean 1.25 mg/day (range: 1–2 mg/day); Max. dose: mean 2.5 mg/day (range: 1–5 mg/day); Duration: n.a.	/	Side effects/tolerability: Patient 1: neutropenia; Psychopathology: improvement in psychopathology from subjective reports; Weight: Improvement in IBW Duration of hospitalization: na
Frank et al., 2017 [39]	N: 22; sex: na; mean age: 15; age range: na	Aripiprazole Min. dose: 1 mg/day; Max dose: 5 mg/day; Duration: na	Comparison groups took part in the same specialized eating disorder inpatient or partial hospital program and were matched for age and had a similar number of inpatient days.	Side effects/tolerability: no ADR reported; Psychopathology: no data were collected Weight: greater improvement in the aripiprazole group; Duration of hospitalization: mean 18.9 days (inpatient); mean 40.4 days (partial hospital treatment).
Tahilioglu et al., 2020 [40]	N: 11; sex: females; mean age: 14; age range: 11–17.	Aripiprazole Min. dose: 0.5 mg/day; Max dose: 15 mg/day; Duration: 540–840 days	/	Side effects/tolerability: no ADR reported; Psychopathology: improvements in obsessive eating attitudes and behaviors in all patients. Increased appetite; change in the CGI-S scores Weight: significant gain; Duration of hospitalization: na
Pimozide				
Plantey, 1977 [41]	N: 1; sex: male; age: 17.	Pimozide Min. dose: 12 mg/day; Duration: 30 days.	/	Side effects/tolerability: no ADR reported; Psychopathology: implacable obsession with weight disappeared; reduction of hyperactivity; Weight: significant gain; Duration of hospitalization: na
Chlorpromazine				
Foster and Kupfer, 1975 [42]	N: 1; sex: female; age: 17.	Chlorpromazine Min. dose: 200 mg/day; Max. dose: 800 mg/day; Duration: 98 days	/	Side effects/tolerability: no ADR reported; Psychopathology: reduction in nocturnal activity; Weight: regained her body weight; Duration of hospitalization: na
Roberts et al., 1986 [43]	N: 1; sex: female; age: 16.	Chlorpromazine Min. dose 25 mg/day; Max dose: 75 mg/day; Duration: na	/	Side effects/tolerability: “grand mal” seizure (direct association unclear potentially mediated by variations in plasma electrolytes). Psychopathology: not directly reported: commented as “reduction of anxiety [...] and fear of eating” in the case report section, in light of the previous literature Weight: significant gain; Duration of hospitalization: na
Roussonis, 1971 [44]	N:1; sex: male; age: 11.	Chlorpromazine Min. dose na; Max dose: na; Duration: na	/	Side effects/tolerability: no ADR reported. Psychopathology: not mentioned; Weight: significant gain; Duration of hospitalization: na
Studies including Multiple Drugs				
Rossi et al., 2007 [45]	N: 19; sex: 17 females, 2 males; mean age: n.a.; age range: 12–18	Haloperidol (n: 4); olanzapine (n: 3); risperidone (n: 1): Min. dose: na *; Max. dose: na *; Duration: mean 150 days; *”low and medium range according to international pharmacological guidelines”	The comparison group has the same characteristics but was treated with SSRIs.	Side effects/tolerability: none 75%, severe drowsiness (12.5%), worsening eating disorder (12.5%); Psychopathology: based on the CGI-S assessment, 75% of the patients showed an improvement in their eating behavior and their psychological status; Weight: BMI increased in all the patients except one (clinical worsening due to haloperidol administration). Duration of hospitalization: n.a.
Ritchie et al., 2009 [46]	N: 1; sex: female; age: 15.	Olanzapine Min. dose: 2.5 mg/die; Max dose: 5 mg/day; Duration: n.a. Risperidone Min. dose: 0.25 mg/day; Max dose 0.75 mg/day; Duration: n.a. Quetiapine Min. dose: 25 mg/day; Max. dose: 50 mg/day; Duration: na;	/	Side effects/tolerability: prolonged QTc (olanzapine and risperidone); Psychopathology: Patient’s anxiety and mood stability improved (quetiapine); Weight: Reestablishment of a healthy weight; Duration of hospitalization: >71 days
Dodig-Ćurković et al., 2010 [47]	N: 1; sex: female; age: 15.	Sulpiride Min. dose 200 mg/day; Duration: na. Olanzapine Min. dose 5 mg/day; Max. dose 15 mg/day Duration: na.	/	Side effects/tolerability: no ADR reported; Psychopathology: decreased delusions of her appearance and body weight; decreased the irrational food selection and caloric values of certain foods; Weight: BMI improvement; Duration of hospitalization: na.

Abbreviations: ADR: Adverse Drug Reactions; N: number; Min: minimum; Max: maximum; n.a.: not available; pt: patient; BMI: Body Mass Index; ED: Eating Disorder; IBW: Ideal Body Weight; RLAI: Risperidone long-acting injection; CGAS: Global Measures of Functioning; CGI-S: Clinical Global Impression scale (Severity of Illness); SSRI: Selective Serotonin Reuptake Inhibitors; CBCL: Child Behavior Checklist; EAT-26: Eating Attitude Test—26 Item; EDI-2: Eating Disorder Inventory-2; SIAB-EX: structured Inventory for Anorexic and Bulimic Eating Disorders; BUT-GSI: Body Uneasiness Test Global Severity Index; BDI-II: Beck Depression Inventory; SAFA-D: Self Administered Psychiatric Scales for Children and Adolescents-Depression; NMS: Neuroleptic Malignant Syndrome.

**Table 2:** Quality assessment of the included studies (case reports excluded). For each included study, the Risk of Bias score and Quality assessment is reported, according to [16,17].

	Drug	Selection	Comparability	Exposure (Case Control)/Outcome (Cohort)	Quality
Spettigue et al., 2018 [20]	Olanzapine	3		2	Poor
Norris et al., 2011 [21]	Olanzapine	3	2	2	Good
Leggero et al., 2010 [22]	Olanzapine	2		1	Poor
Rossi et al., 2007 [45]	Haloperidol, risperidone, olanzapine	2		1	Poor
Frank et al., 2017 [39]	Aripiprazole	4	2	1	Poor
Pruccoli et al., 2022 [24]	Olanzapine	2	2	2	Fair
Tahıllıoğlu et al., 2020 [40]	Aripiprazole	1		2	Poor

**Table 3:** Available evidence for female and male patients.

	Females		Males	
	Quality	Summary of Findings	Quality	Summary of Findings
Olanzapine	10 studies (1 prospective study, 1 retrospective study, 3 case series, 5 case reports)	− weight gain (9/10), weight restored (1/10) − no improvement in psychopathology (1/10); remission of psychiatric symptoms: irritability (2/10), anxiety (3/10), agitation (3/10), compulsive thoughts about weight, food, and physical activity (6/10) − excessive sedation (1/10); mild sedation (3/10); elevations in the fasting lipid profile; elevated liver function test (2/10); itching (1/10); no ADR reported (6/10)	1 case report	− no improvement in weight − no improvement in psychopathology − NMS occurred 48 h after the first administration
Risperidone	3 case reports	− weight gain (3/3) − improvement in psychopathology (3/3): less rigidity during meal timing (1/3); more insight about anorexia and lowered anxiety (1/3); decrease in agitation and aggression, loss of paranoid ideation, more focused attention (1/3) − no ADR reported	1 case report	− weight gain − reduction of agitation during enteral feeding; reduction in body distortion − refusal of the oral formulation
Aripiprazole	2 case series	− weight gain (2/2) − psychopathology improvement (in obsessive eating attitudes and behaviors 1/2) − neutropenia in 1 patient	/	
Quetiapine	1 case report	− weight gain − improved in anxiety and mood stability − no ADR reported	/	
Clotiapine	1 case report	− no weight information reported − decrease of delusional symptoms; − fatigue (1 patient)	/	
Chlorpromazine	2 case reports	− weight restored (2/2) − scarce data on psychopathology − one grand mal seizure (association unclear)	1 case report	− weight restored − increased caloric intake − no ADR reported
Haloperidol				
Pimozide	/		1 case report	− weight restored − decrease in obsessive thoughts and hyperactivity − no ADR reported
Sulpiride	1 case report	− weight gain − decrease in distorted self-image and thoughts around food − no ADR reported	/	

**Table 4:** Available evidence for age groups.

	Children (<14 years)		Adolescents (≥14 years)	
	Quality	Summary of Findings	Quality	Summary of Findings
Olanzapine	2 case reports/series	− weight gain (1/2); weight restored (1/2) − no ADR reported − reduced rituals and compulsions (2/2); improvement in anxiety and agitation (1/2); better in sleeping (1/2)	5 case reports	− reduction in abnormal eating habits (2/5); anxiety and agitation (1/5); irritability, compulsive thoughts about body weight and physical activity; no data collected (1/5); no improvement (1/5) − weight gain (4/5); no improvement (1/5) − no ADR reported (3/5); mild sedation (1/5); NMS (1/5)
Risperidone	3 case reports	− weight gain − no ADR reported (2/3); refusal oral formulation (1/3) − decrease in agitation (2/3); improvement in paranoid and distorted thoughts (2/3); cheerful and energetic (1/3)	1 case report	− weight gain − improvement in rigidity during mealtime − no ADR reported
Aripiprazole	/		/	
Quetiapine	/		1 case report	− weight gain − improved in anxiety and mood stability − no ADR reported
Clotiapine	/		1 retrospective chart review	− weight: na − decrease delusional thinking and restored proper hygiene habits − fatigue
Chlorpromazine	2 case reports	− weight restored (2/2) − scarce data on psychopathology − one grand mal seizure (association unclear)	1 case report	− weight restored − increased caloric intake − no ADR reported
Haloperidol	/		/	−
Pimozide	/		1 case report	− weight gain − reduction in hyperactivity and obsessive thoughts about weight − no ADR reported
Sulpiride	/		1 case report	− weight gain − decrease in distorted self-image and thoughts around food − no ADR reported

## Data Availability

Not applicable.
